# Spatiotemporal and Functional Heterogeneity of Hematopoietic Stem Cell-Competent Hemogenic Endothelial Cells in Mouse Embryos

**DOI:** 10.3389/fcell.2021.699263

**Published:** 2021-08-11

**Authors:** Yun-Qiao Li, Yandong Gong, Siyuan Hou, Tao Huang, Haizhen Wang, Di Liu, Yanli Ni, Chaojie Wang, Junliang Wang, Jun Hou, Ruichuang Yang, Jing Yan, Guangyu Zhang, Bing Liu, Yu Lan

**Affiliations:** ^1^State Key Laboratory of Proteomics, Academy of Military Medical Sciences, Academy of Military Sciences, Beijing, China; ^2^State Key Laboratory of Experimental Hematology, Fifth Medical Center of Chinese PLA General Hospital, Institute of Hematology, Beijing, China; ^3^Key Laboratory for Regenerative Medicine of Ministry of Education, School of Medicine, Institute of Hematology, Jinan University, Guangzhou, China; ^4^Peking-Tsinghua Center for Life Sciences, Peking University, Beijing, China; ^5^Department of Radiotherapy, The Fifth Medical Center of Chinese PLA General Hospital, Beijing, China; ^6^The Fifth Medical Center of PLA General Hospital, National Clinical Research Center for Infectious Diseases, Beijing, China

**Keywords:** hematopoietic stem cells, hemogenic endothelial cells, heterogeneity, developmental hematopoiesis, single-cell RNA-sequencing

## Abstract

Hematopoietic stem cells (HSCs) are derived from hemogenic endothelial cells (HECs) during embryogenesis. The HSC-primed HECs increased to the peak at embryonic day (E) 10 and have been efficiently captured by the marker combination CD41^–^CD43^–^CD45^–^CD31^+^CD201^+^Kit^+^CD44^+^ (PK44) in the aorta-gonad-mesonephros (AGM) region of mouse embryos most recently. In the present study, we investigated the spatiotemporal and functional heterogeneity of PK44 cells around the time of emergence of HSCs. First, PK44 cells in the E10.0 AGM region could be further divided into three molecularly different populations showing endothelial- or hematopoietic-biased characteristics. Specifically, with the combination of Kit, the expression of CD93 or CD146 could divide PK44 cells into endothelial- and hematopoietic-feature biased populations, which was further functionally validated at the single-cell level. Next, the PK44 population could also be detected in the yolk sac, showing similar developmental dynamics and functional diversification with those in the AGM region. Importantly, PK44 cells in the yolk sac demonstrated an unambiguous multilineage reconstitution capacity after *in vitro* incubation. Regardless of the functional similarity, PK44 cells in the yolk sac displayed transcriptional features different from those in the AGM region. Taken together, our work delineates the spatiotemporal characteristics of HECs represented by PK44 and reveals a previously unknown HSC competence of HECs in the yolk sac. These findings provide a fundamental basis for in-depth study of the different origins and molecular programs of HSC generation in the future.

## Introduction

As the cornerstone of the adult hematopoietic system, self-renewal and multilineage hematopoietic differentiation are the dominant features of hematopoietic stem cells (HSCs). HSCs are widely proposed to be derived from a special subgroup of endothelial cells, called hemogenic endothelial cells (HECs), in the aorta-gonad-mesonephros (AGM) region around embryonic day (E) 10.0 through the endothelial-to-hematopoietic transition (EHT) process. It is widely proposed that HECs express CD47, CD61, and Dll4 (Huang et al., 2016; Zhou et al., 2016) in addition to pan-endothelial markers such as Flk1 and VE-Cadherin (Yamashita et al., 2000; Yokomizo and Dzierzak, 2010; Hadland et al., 2017) but lack the expression of hematopoietic markers CD41, CD43, and CD45 (Taoudi et al., 2005; Swiers et al., 2013). The expression of Runx1, a transcription factor critically required for the process of EHT (Chen et al., 2009; Swiers et al., 2013; Gao et al., 2018; Lie et al., 2018), represents a hallmark of HECs. Based on this, *Runx1* + *23GFP* (GFP transgene on the Runx1 + 23 enhancer locus) transgenic reporter mouse model has been established to enrich HECs for molecular/phenotypic and functional analyses (Swiers et al., 2013). Similarly, *Gfi1-Tomato* transgenic mice are used to delineate the process of EHT (Thambyrajah et al., 2016). However, the enrichment with the surface markers or transgenic reporters mentioned above is insufficient, and the HSC competence of HECs has not been directly confirmed.

Recently, we elucidated the precise identity of HSC-primed HECs by high-precision single-cell transcriptomic profiling. With *in vivo* functional validation, we identified the PK44 (CD41^–^CD43^–^CD45^–^CD31^+^CD201^+^Kit^+^CD44^+^) population as HSC-competent HECs (Hou et al., 2020). Additionally, functional heterogeneity of the PK44 population was revealed *in vitro* at the single-cell level, involving three kinds of differentiation potentials, namely, only endothelial potential, endothelial-hematopoietic dual potential, and only hematopoietic potential (Hou et al., 2020). Nevertheless, the characteristics of these heterogeneous subpopulations within immunophenotypic PK44 cells remain unclear and deserve further investigation.

Whether the hematopoietic cells produced by the extra-embryonic yolk sac can sustain hematopoiesis during the lifespan has long been debated (Samokhvalov et al., 2007; Lee et al., 2016). The blood circulation in embryos precludes the possibility of precisely addressing if the yolk sac can *in situ* produce HSCs (Cumano et al., 2001). It is known that HECs also exist in the extra-embryonic yolk sac besides the intra-embryonic AGM region (Frame et al., 2016; Lee et al., 2016). Considering that PK44 efficiently enriches HSC-primed HECs in AGM, the performance of the immunophenotypic PK44 population in the yolk sac is worthy of investigation.

Index sorting is a very innovative mode of flow cytometry that can capture the expression profiles of multiple proteins of each sorted cell (Schulte et al., 2015). The characteristics of index sorting make it useful in exploring immunophenotypes of populations with a specific functional readout. In this study, we focus on the hematopoietic potential of PK44 cells, and we achieved further enrichment of the hematopoietic potential within PK44 cells in the AGM region using additional surface markers by the index sorting function. We also explored the spatiotemporal and functional heterogeneity of AGM and yolk sac PK44 populations. Our findings in total shed light on the in-depth understanding of HSC emergence during embryogenesis.

## Materials and Methods

### Mice

All the mice used in the experiment were raised in the Laboratory Animal Center of Academy of Military Medical Sciences. The experimental operation of mice was carried out with the approval of the Animal Care and Use Committee of the Institute. All mice were maintained on C57BL/6 background. Embryos were staged by somite pair (sp) counting: E9.5, 21-30 sp; E10.0, 31-35 sp; E10.5, 36-40 sp; and E11.0, 41-45 sp. For E9.5 embryos, the caudal half was dissected under the heart with the limbs removed. For embryos older than E10.0, the AGM region was dissected as previously reported (Hou et al., 2020).

### Flow Cytometry

Cells were analyzed and sorted by flow cytometers FACS Aria 2 and Calibur (BD Biosciences), and the data were analyzed with FlowJo software (Tree Star). Cells were stained by the following antibodies: B220 (eBioscience, RA3-6B2), CD3 (eBioscience, 145-2C11), CD4 (eBioscience, GK1.5), CD8 (eBioscience, 53-6.7), CD19 (eBioscience, eBio1D3), CD31 (BD or BioLegend, MEC13.3), CD34 (eBioscience, RAM34), CD41 (BD or eBioscience, MWReg30), CD43 (BD, S7), CD44 (eBioscience or BioLegend, IM7), CD45 (eBioscience, 30-F11), CD45.1 (eBioscience, A20), CD45.2 (eBioscience, 104), CD47 (eBioscience, miap301), CD49d (Biolegend, R1-2), CD93 (eBioscience, AA4.1), CD133 (Biolegend, 315-2C11), CD146 (BD, ME-9F1), CD201 (eBioscience, eBio1560), CD304 (Biolegend, 3E12), Flk1 (eBioscience, Avas12a1), Flk2 (Biolegend, A2F10), F4/80 (Biolegend, 93), Kit (eBioscience, 2B8), Ly-6G (BioLegend, 1A8), Mac-1 (eBioscience, M1/70), Sca-1 (eBioscience, D7), Ter119 (BD, TER-119), and 7-amino- actinomycin D (7-AAD; eBioscience).

### Colony Forming Unit-Culture (CFU-C) Assay

Cells were sorted by flow cytometry and cultured in a 35 mm Petri dish containing 2.5 mL methylcellulose-based medium with recombinant cytokines (MethoCult GF M3434, STEMCELL Technologies) for 7 days for the colony-forming assay. Hematopoietic colonies were scored individually by microscope.

### *In vitro* Hematopoietic and Endothelial Potential Assay

Cells were sorted by the FACS Diva 8 “index sorting” function in single-cell mode and were then individually plated on OP9-DL1 stromal cells in IMDM (Hyclone) containing 1% bovine serum albumin (Sigma), 10 μg/mL insulin (Macgene), 15% fetal bovine serum (Hyclone), 5.5 × 10^–5^ mol/L 2-mercaptoethanol (Gibco), and 200 μg/mL transferrin (Sigma), 50 ng/mL SCF (PeproTech) and 100 ng/mL rhVEGF-165 (PeproTech). After 7 days co-culture with OP9-DL1, cells were fixed in 2% paraformaldehyde for 20 min and then stained with CD45 antibody (BD Biosciences). Subsequently, CD31 (BD Pharmingen, MEC13.3) immunohistochemistry staining was performed. When only CD31^+^ endothelial tubes were detected, the single cell plated in the well was considered to have only endothelial potential. When CD45^+^ round cells but no CD31^+^ endothelial tubes were detected, the single cell plated in the well was considered to have only hematopoietic potential. When both CD31^+^ endothelial tubes and CD45^+^ round cells were simultaneously detected in the same single-cell well, the plated cell was considered to have both endothelial and hematopoietic potential (Hou et al., 2020).

### OP9-DL1 Co-culture and HSC Transplantation Assay

To investigate the HSC potential of the PK44 population in E10.0 AGM and yolk sac, male CD45.1/1 mice were mated with female CD45.2/2 mice to obtain CD45.1/2 embryos, which were used as donors for PK44 cells. PK44 cells were sorted and plated on OP9-DL1 stromal cells in α-MEM (Gibco) containing 10% fetal bovine serum (Hyclone), 2 mM glutamine (Hyclone), 100 U/mL penicillin, and 100 mg/mL streptomycin (Hyclone) and cytokines (100 ng/mL SCF, 100 ng/mL Flt3 ligand, and 100 ng/mL IL-3, all from PeproTech) as we previously reported (Hou et al., 2020). After 7 days of co-culture, all cells in each well were collected (with trypsin digestion), and the culture progenies together with 2 × 10^4^ nucleated fresh bone marrow carrier cells (CD45.1/1 background) were injected into 10- to 12-week female recipients (CD45.2/2 background), which were exposed to a split dose of 9 Gy γ-irradiation (^60^Co) via tail vein. Peripheral blood cells of recipients were detected by flow cytometry at 4, 8, 12, and 16 weeks post-transplantation, respectively, to determine the chimerism. The recipients demonstrating ≥5% donor-derived chimerism in CD45^+^cells of peripheral blood were counted as successfully reconstituted. Multiorgan and multilineage reconstitution were analyzed as reported (Hou et al., 2020).

### Preprocessing Single-Cell RNA-Seq Data

For scRNA-seq data from modified STRT-seq, raw reads of each cell were first split by a specific barcode sequence attached in Read 2. The template switch oligo (TSO) sequence and polyA tail sequence were trimmed for the corresponding Read 1 after UMI information was aligned to it. Subsequently, reads with adapter contaminants or low-quality bases (N > 10%) were discarded. Next, the stripped Read 1 sequences were aligned to mm10 mouse transcriptome (UCSC) using Hisat2 (version 2.1.0) (Kim et al., 2015). Uniquely mapped reads were counted by HTSeq package (Anders et al., 2015) and grouped by the cell-specific barcodes. Duplicated transcripts were removed based on the UMI information for each gene. Finally, for each individual cell, the copy number of transcripts of a given gene was the number of the distinct UMIs of that gene. To filter low-quality cells, count values for each cell were first grouped in an expression matrix, and only cells with more than 2000 genes and 10,000 transcripts detected were retained. Also, cells with too many raw reads (> 1,000,000) and genes (> 10,000) were excluded because these cells might not be real single cells.

### Cell Type Detection and Dimensionality Reduction

Downstream analysis for well-based modified STRT-seq—such as data normalization, clustering, differential expression analysis, principal component analysis (PCA), and Uniform Manifold Approximation and Projection (UMAP) analysis—were implemented using the R package Seurat 2 and monocle 2. Harmony was employed to integrate AGM and yolk sac data.

### Differentially Expressed Genes (DEGs) and Cluster Biomarker Identification

DEGs were identified by running the “FindAllMarkers” function using ROC test (Figure 2) or Wilcoxon rank sum test (Figure 5) in Seurat 2. High-confidence surfaceome proteins identified in the Cell Surface Protein Atlas were marked as surface molecules. All DEGs of specific clusters and the surface proteins list are listed in the Supplementary Tables 1, 2.

### Gene Ontology (GO) Analysis

To interpret the biological meaning of DEGs, GO analyses were performed by clusterProfiler using all the DEGs, and then, the top 10 GO terms with *P* value < 0.05 were selected for visualization using bar plots.

### Endothelial and Hematopoietic Feature Score

The endothelial and hematopoietic feature score was assigned to each cell by using the “AddModuleScore” function in Seurat. Fifteen representative endothelial genes (*Gja4*, *Unc5b*, *Mecom*, *Hey1*, *Efnb2*, *Dll4*, *Vegfc*, *Epas1*, *Cxcr4*, *Igfbp3*, *Nr2f2*, *Aplnr*, *Nrp1*, *Nrp2*, *Pecam1*) (Chong et al., 2011; Corada et al., 2014; Simons and Eichmann, 2015; Su et al., 2018) and eight representative hematopoietic genes (*Adgrg1*, *Ikzf2*, *Bcl11a*, *Runx1*, *Neurl3*, *Angpt1*, *Gfi1*, *Hlf*) (Zhou et al., 2016; Hou et al., 2020) were selected to calculate the endothelial and hematopoietic feature score for each cell, respectively.

### Statistical Analysis

All statistical analyses were conducted in R version 3.5.0. ROC analysis and two sample Wilcoxon rank sum tests were employed for comparisons of gene expression levels between two clusters of cells. GO biological process enrichment analyses were performed using clusterProfiler. We referred to statistically significant as *P* < 0.05 (if not specified).

## Results

### Transcriptomically Different Subpopulations in AGM PK44 Cells

**FIGURE 1:**
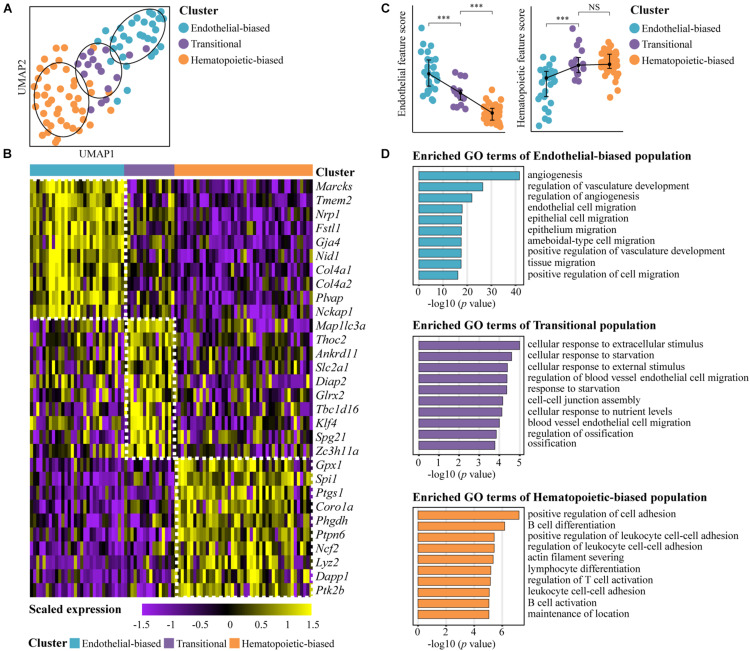
Transcriptomically different subpopulations in E10.0 AGM PK44 cells. **(A)** UMAP plots of the E10.0 AGM PK44 cells with clusters mapped onto it. **(B)** Heatmap showing top 10 DEGs of three subpopulations (endothelial-biased, transitional, and hematopoietic-biased population) in E10.0 AGM PK44 cells. DEGs were sorted by ROC power calculated through FindallMarkers function (test.use = “roc”) in Seurat2. **(C)** Dot plot showing the endothelial and hematopoietic scores of E10.0 AGM PK44 cells in three subpopulations to, respectively, represent the endothelial and hematopoietic characteristics molecularly. The statistical significance of differences was determined using Wilcox test. ^∗∗∗^*P* < 0.001. **(D)** Enriched GO terms of three subpopulations in E10.0 AGM PK44 cells and top 10 terms with *P* value < 0.05 are shown.

According to a graph-based unsupervised clustering approach from Seurat software, 93 E10.0 AGM PK44 cells from our previous scRNA-seq dataset that had passed rigorous quality control were separated into three clusters (Hou et al., 2020) (Figure 1A). The endothelial-biased subpopulation was readily recognized by the obvious *Nrp1* and *Gja4* expression (Chong et al., 2011; Corada et al., 2014; Simons and Eichmann, 2015; Su et al., 2018). The hematopoietic-biased cluster was featured by its relatively high expression of *Spi1* (Thambyrajah et al., 2016; Hou et al., 2020). The remaining one was named the transitional population given its moderate expression level of endothelial and hematopoietic genes (Figure 1A–C). Then, the three subpopulations were analyzed by Gene Ontology, and it was found that the endothelial-biased subpopulation enriched genes were mainly involved in angiogenesis and regulation of vasculature, and the genes enriched in hematopoietic-biased subpopulation were mainly related to positive regulation of cell adhesion, B cell differentiation, and regulation of T cell activation (Figure 1D). Taken together, these results suggested the existence of molecular heterogeneity within AGM PK44 population.

### Further Enrichment of Hematopoietic Potential by Additional Marker Within AGM PK44 Cells

**FIGURE 2:**
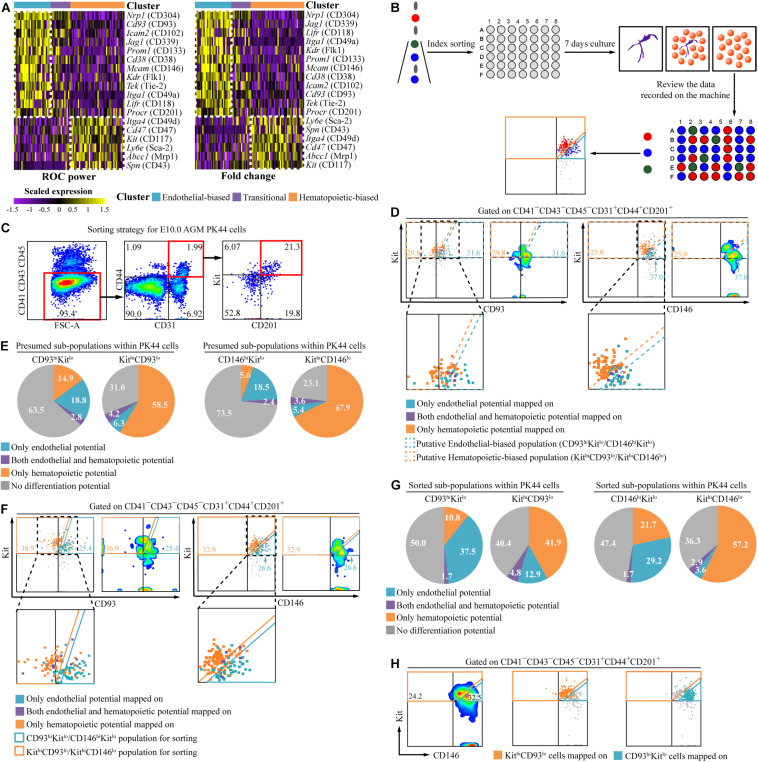
Functionally different subpopulations within E10.0 AGM PK44 cells. **(A)** Heatmap showing the differentially expressed surface marker coding genes of the subpopulations in E10.0 AGM PK44 cells arranged by ROC power (left) and fold change (right), respectively. For the convenience of FACS labeling and live cell sorting, only genes coding cell surface proteins whose antibodies for FACS labeling are also commercially available in China within the DEG list are displayed. **(B)** Schematic experimental design of screening candidate molecules by index sorting. **(C)** Flow cytometry gating strategy for isolating PK44 population (CD41^–^ CD43^–^ CD45^–^ CD31^+^CD44^+^ Kit^+^CD201^+^) from the E10.0 AGM region. **(D)** FACS plots showing expression of Kit and CD93/CD146 on the index-sorted E10.0 AGM PK44 cells. Cells with different kinds of potentials based on *in vitro* functional evaluation (color dots) are mapped onto the reference FACS plots (gray dots). Data are from four independent experiments. The enlarged views of dashed black boxes are shown below. **(E)** Pie charts showing the proportions of four differentiation potentials in the putative subpopulations. **(F)** FACS plots showing expression of Kit and CD93/CD146 on the index-sorted candidate subpopulations of E10.0 AGM PK44 cells. Cells with different potentials based on *in vitro* functional evaluation (color dots) are mapped onto the reference FACS plots (gray dots). Data are from four independent experiments. The enlarged views of dashed black boxes are shown below. **(G)** Pie charts showing the proportions of four differentiation potentials in each of the isolated subpopulations. **(H)** FACS plots of Kit and CD146 expression with the indicated subpopulations mapped on. Orange boxes indicate the gates of the Kit^hi^CD146^lo^ subpopulation; cyan boxes indicate the gates of the CD146^hi^Kit^lo^ subpopulation.

Based on the transcriptomic analysis, we explored whether the endothelial- or hematopoietic-biased subpopulation could be further enriched by additional surface markers. First, we ranked the differentially expressed surface markers according to ROC power and fold change for antibody screening (Figure 2A). Among these molecules, endothelial markers *Nrp1*, *Kdr*, and *Tek* were highly expressed in the endothelial-biased subpopulation (Shalaby et al., 1997; Takakura et al., 1998; Yamashita et al., 2000; Simons and Eichmann, 2015), whereas *CD47* and *Kit* were overrepresented in the hematopoietic-biased subpopulation (North et al., 2002; Taoudi et al., 2005; Zhou et al., 2016; Hou et al., 2020). No surface marker was screened out being highly expressed in the transitional subpopulation (Figure 2A).

Next, we tried several predicated surface markers in the following functional assays, including the ones highly expressed in the endothelial-biased subpopulation, CD304, Flk1, CD93, and CD146, and those highly expressed in the hematopoietic-biased subpopulation, CD47 and CD49d (Figure 2A). We, respectively, added each of these selected surface markers to PK44 combination and used the index sorting function to sort individual PK44 cells into 48-well plates with one cell per well (Figures 2B,C). After co-culture with OP9-DL1 for 7 days, the cultures were stained with CD45 and CD31 to determine the presence of hematopoietic clusters and endothelial tubes, respectively (Supplementary Figure 1A) (Hou et al., 2020). The number and proportion of cells with only endothelial potential, endothelial-hematopoietic dual potential, and only hematopoietic potential were counted (Supplementary Figure 1A), and these were generally in line with that in our previous report (Hou et al., 2020). By retrospectively analyzing the corresponding expression of the selected markers on each cell via index sorting data (Figure 2B), we identified CD93 and CD146 as candidates, which could further distinguish the cells with different potentials within PK44 population (Figures 2D,E and Supplementary Figure 1B), and excluded Flk1, CD304, CD47, and CD49d given their poor performance in further discriminating endothelial- or hematopoietic-biased subpopulations (Supplementary Figure 1D). In detail, cells with different potentials were basically separated on the plots when displaying the expression of Kit and one other candidate (CD93 or CD146) (Figure 2D). The cells with hematopoietic potential showed relatively high expression of Kit and low expression of the two candidates (CD93 or CD146) and were, thus, abbreviated as Kit^hi^CD93^lo^ and Kit^hi^CD146^lo^. On the other hand, the endothelial potential was concentrated in the subpopulation with relatively high expression of the candidates (CD93 or CD146) and low expression of Kit (abbreviated as CD93^hi^Kit^lo^ and CD146^hi^Kit^lo^) (Figures 2D,E and Supplementary Figures 1B,E).

To determine whether these two combinations could prospectively enrich potential-biased subpopulations, we, respectively, sorted Kit^hi^CD93^lo^, CD93^hi^Kit^lo^, Kit^hi^CD146^lo^, and CD146^hi^Kit^lo^ subpopulations within the PK44 population according to their expression patterns on FACS for *in vitro* single-cell functional evaluation (Figure 2F). When the differentiation potential of these cells was retrospectively projected on the FACS plots, the bias of potential for the subpopulations was clearly observed (Figures 2F,G). In detail, the proportions of cells with hematopoietic progenies in the Kit^hi^CD93^lo^ (52/124 ≈ 41.9%) and Kit^hi^CD146^lo^ (79/138 ≈ 57.2%) subpopulations were about 3.9- and 2.6-fold higher than those in the CD93^hi^Kit^lo^ (13/120 ≈ 10.8%) and CD146^hi^Kit^lo^ (26/120 ≈ 21.7%) subpopulations, respectively (Figure 2G and Supplementary Figures 1C,F). Compared with that of PK44 population without subdividing (Supplementary Figure 1A), the enrichment efficiency of the Kit^hi^CD93^lo^ and Kit^hi^CD146^lo^ combinations for the hematopoietic potential increased about 1.3- and 1.7-fold, respectively (Supplementary Figure 1F). Expectedly, the similarity between Kit&CD93 and Kit&CD146 combinations to discriminate two potential-biased subpopulations was observed (Figure 2H). Interestingly, both endothelial- and hematopoietic-biased subpopulations, either index-sorted or prospectively isolated, comprise a small proportion of cells with endothelial-hematopoietic dual potential (1.7%–4.8%), which stood for a transient intermediate state of the cells undergoing fate choice (Hou et al., 2020) (Figures 2E,G). The existence of this rare potential suggests that the PK44 population is a continuum for the EHT process. Collectively, we further caught the subpopulations with either endothelial- or hematopoietic-biased characteristics by markers screened out from transcriptomic data.

### Developmental Dynamics of the Immunophenotypic PK44 Cells in AGM and Yolk Sac

**FIGURE 3:**
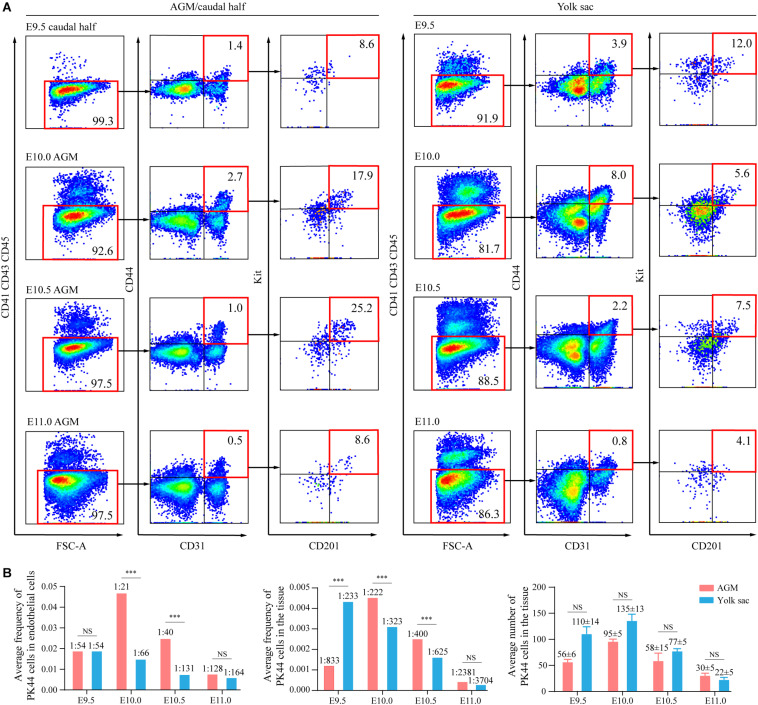
Developmental dynamics of the immunophenotypic PK44 cells in AGM and yolk sac. **(A)** Representative FACS plots for AGM/caudal half and yolk sac PK44 cells from E9.5 to E11.0. **(B)** Graphs showing the proportions of PK44 cells in endothelial cells (CD41^–^ CD43^–^ CD45^–^ CD31^+^) (left) and in total cells of the corresponding tissues (middle). The statistical significance of differences was determined using Fisher’s exact test. ^∗∗∗^*P* < 0.001. NS, not significant. The average numbers of PK44 cells in corresponding tissues are shown to the right. Data are means ± SD. Data are from three independent experiments. The statistical significance of differences was determined using Mann–Whitney test. NS, not significant.

We then analyzed the spatiotemporal expression characteristics of the immunophenotypic PK44 populations in AGM and yolk sac from E9.5 to E11.0 at an interval of half a day, around the time of emergence of HSCs (Figure 3A). The proportion of the PK44 population in the AGM/caudal half region increased from E9.5 to a peak at E10.0, reaching 0.45% (1:222), and then decreased gradually. In comparison, this proportion in the yolk sac was highest at E9.5, reaching 0.43% (1:233) (Figures 3A,B). For absolute quantities, both AGM and yolk sac PK44 populations reached the peak at E10.0, which were 95 ± 5 and 135 ± 13 and then met a gradual drop (Figure 3B). The previously unknown expression profile of the immunophenotypic PK44 cells in yolk sac provide a basis for the following *in vitro* and *in vivo* functional experiments.

### Functional Differences of the Immunophenotypic PK44 Cells Between AGM and Yolk Sac

**FIGURE 4:**
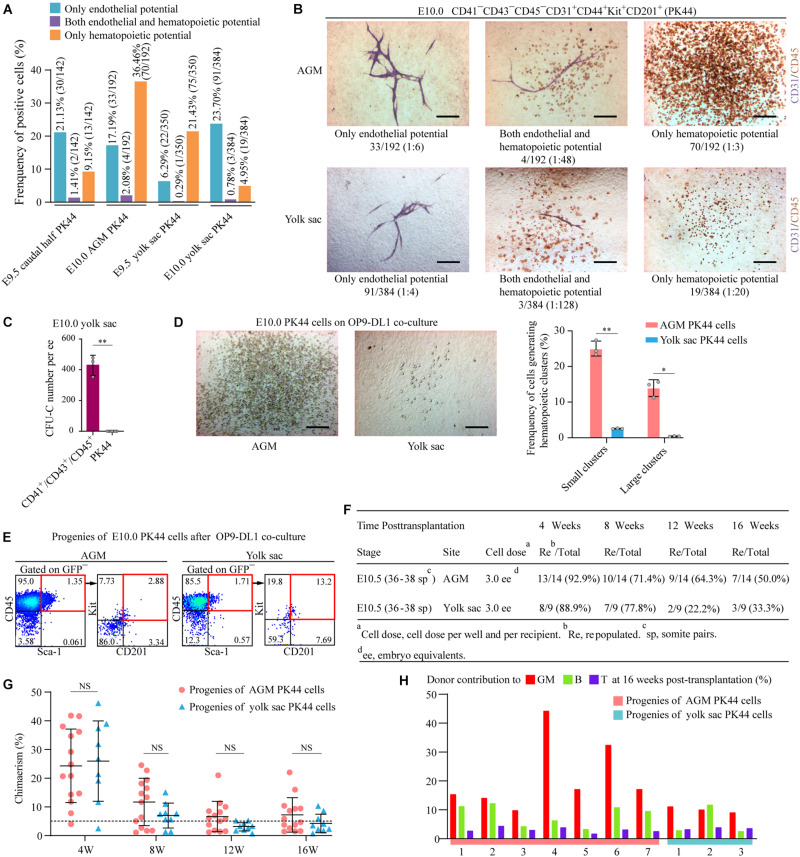
Functional evaluation of yolk sac PK44 cells. **(A)** Column charts showing the frequencies of potential-positive cells in the indicated populations for each kind of differentiation potentials. Data are from three independent experiments. **(B)** Representative CD31 and CD45 immunostaining on the cultures of single PK44 cells from E10.0 AGM and yolk sac, showing typical morphologies regarding distinct differentiation potentials. Cell frequencies of each kind of potentials are shown. Data are from three independent experiments. Scale bars, 100 μm. **(C)** Number of hematopoietic progenitors per embryo equivalent (ee) in the indicated populations derived from E10.5 yolk sac measured by the methylcellulose CFU-C assay. Data are means ± SD. The statistical significance of differences was determined using the Wilcox test. ^∗∗^*P* < 0.01. Data are from three independent experiments. **(D)** Morphology of hematopoietic progenies generated by AGM (left) and yolk sac (middle) PK44 cells after OP9-DL1 co-culture for 5 days. Scale bars, 100 μm. Column charts in the right showing the frequencies of AGM and yolk sac PK44 cells generating small or large hematopoietic clusters. Data are from three independent experiments. Data are means ± SD. The statistical significance of differences was determined using Pearson’s chi-squared test. ^∗^*P* < 0.05; ^∗∗^*P* < 0.01. **(E)** Representative FACS plots of analyzing the progenies of E10.0 AGM and yolk sac PK44 cells following 7 days co-culture, showing a population of cells expressing putative HSC markers. **(F)** Detailed information of the transplantation assays performed with the progenies of E10.5 AGM and yolk sac PK44 cells. **(G)** Graphs showing the donor chimerism from 4 to 16 weeks after being transplanted with the progenies of the E10.5 AGM and yolk sac PK44 populations (three embryo equivalents per recipient), respectively. Data are means ± SD. The statistical significance of differences was determined using Mann–Whitney test. NS, not significant. **(H)** Bar plots showing the donor contribution to the granulocytes/monocytes (GM, red), B lymphocytes (green), and T lymphocytes (purple) in the peripheral blood of each of the primary recipients receiving the progenies of E10.5 AGM and yolk sac PK44 cells at 16 weeks post-transplantation.

With the aim of further exploring whether the yolk sac PK44 cells are HECs transitioning into hematopoietic fate and have endothelial-hematopoietic dual potential like that in the AGM region, we first performed the *in vitro* hematopoietic and endothelial differentiation assays at the single-cell level of E9.5 and E10.0 yolk sac PK44 cells with E9.5 caudal half and E10.0 AGM PK44 cells as positive controls. Interestingly, yolk sac PK44 cells also possessed the three differentiation potentials, namely, only endothelial potential (E9.5: 22/350 ≈ 6.29%, E10.0: 91/384 ≈ 23.70%), endothelial-hematopoietic dual potential (E9.5: 1/350 ≈ 0.29%, E10.0: 3/384 ≈ 0.78%), and only hematopoietic potential (E9.5: 75/350 ≈ 21.43%, E10.0: 19/384 ≈ 4.95%) (Figures 4A,B). From E9.5 to E10.0, the proportion of cells with hematopoietic potential in yolk sac PK44 cells sharply decreased in contrast to that in the intra-embryonic PK44 cells which showed an obvious increase (Figure 4A). The methylcellulose-based culture system supports hematopoietic differentiation from hematopoietic progenitors rather than blood generation from endothelial precursors. Similar to AGM PK44 cells (Hou et al., 2020), yolk sac PK44 cells did not form hematopoietic colonies in methylcellulose (Figure 4C), confirming their identity as endothelial cells rather than hematopoietic progenitors. Using index sorting data, we retrospectively analyzed the expression level of CD201, Kit, and CD44 of E9.5 yolk sac PK44 cells whose potential had been individually determined and found that no paired combinations of the three markers could effectively distinguish hematopoietic or endothelial potentials from one another (Supplementary Figure 2).

We next compared the hematopoietic potential of E10.0 AGM and yolk sac PK44 cells after co-cultured on OP9-DL1 under the culture condition for inducing HSCs (Zhou et al., 2016; Hou et al., 2020). Through morphological observation, a large hematopoietic cluster was defined as the one with diameter greater than or equal to 1.5 mm (McNiece et al., 1990) after 5 days co-culture. Under this criteria, yolk sac PK44 cells generated fewer hematopoietic clusters (yolk sac: 58/1729≈ 3.4%; AGM: 184/465 ≈ 39.6%) with a lower proportion of large ones (yolk sac: 10/58 ≈ 17.2%; AGM: 66/184 ≈ 35.9%) than AGM PK44 cells (Figure 4D). After co-culture for 7 days, the hematopoietic products in the cultures were assessed by FACS. Although the total hematopoietic cells generated by PK44 cells from yolk sac were much fewer than those from AGM (Figure 4D), both AGM and yolk sac PK44 cells were capable of giving rise to CD45^+^Sca-1^+^Kit^+^CD201^+^ immunophenotypic HSPCs, suggesting the yolk sac PK44 population as a potential origin for HSPCs (Figure 4E).

The *in vivo* repopulating capacity of the progenies of PK44 cells was further evaluated, and the multilineage reconstitution was found in 7/14 and 3/9 recipient mice 16 weeks after being transplanted with the culture products of AGM and yolk sac PK44 cells, respectively (Figures 4F–H and Supplementary Figure 3A). Moreover, the donor-derived immunophenotypic HSCs were detected in the bone marrow of recipients transplanted with the progenies of PK44 cells from both AGM and yolk sac (Supplementary Figures 3B,C). This finding revealed a previously unknown HSC competence of HECs represented by the PK44 population in the yolk sac around the time for HSC emergence during embryogenesis.

### Analysis of Transcriptomic Characteristics of Yolk Sac PK44 Cells

**FIGURE 5:**
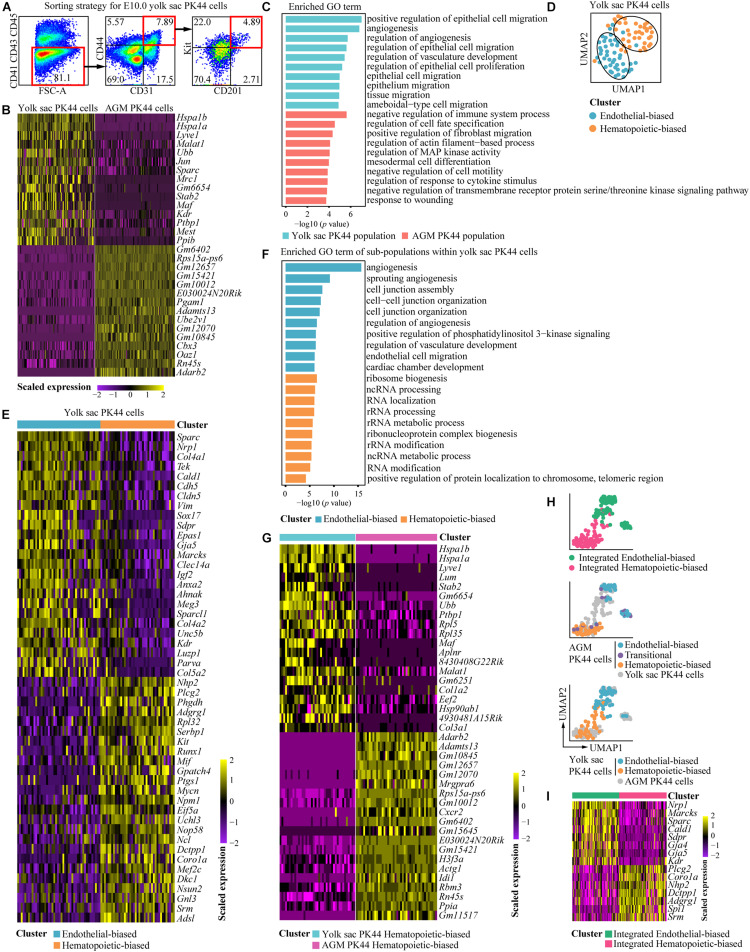
Transcriptomic characteristics of E10.0 yolk sac PK44 cells. **(A)** Flow cytometry gating strategy for isolating PK44 cells in E10.0 yolk sac. **(B)** Heatmap showing the top 15 DEGs between E10.0 yolk sac and AGM PK44 cells. **(C)** Enriched GO terms of E10.0 yolk sac and AGM PK44 populations. **(D)** UMAP plots of the E10.0 yolk sac PK44 cells with clusters mapped on. **(E)** Heatmap showing top 25 DEGs between two subpopulations (endothelial- and hematopoietic-biased) in E10.0 yolk sac PK44 cells. **(F)** Enriched GO terms of two subpopulations (endothelial- and hematopoietic-biased) in E10.0 yolk sac PK44 cells. **(G)** Heatmap showing top 20 DEGs between hematopoietic-biased subpopulation in E10.0 yolk sac PK44 cells and that in E10.0 AGM PK44 cells. **(H)** UMAP of integrated data of E10.0 AGM and yolk sac PK44 cells with subpopulations (endothelial-biased, transitional, and hematopoietic-biased population) mapped on. **(I)** Heatmap showing DEGs between the integrated endothelial- and hematopoietic-biased populations.

To further investigate the molecular characteristics of the yolk sac PK44 population, we used STRT-seq to perform single-cell transcriptomic profiling of E10.0 yolk sac PK44 cells (Figure 5A). Of the 87 cells that passed the strict quality control, we detected, on average, 5139 genes and 248,722 transcripts for each cell. Of note, the well-known yolk sac endothelial markers *Lyve1* and *Stab2* were highly expressed in the PK44 cells from the yolk sac compared with those from AGM region (Nonaka et al., 2007; Lee et al., 2016; Ganuza et al., 2018; Shvartsman et al., 2019) (Figure 5B). The genes enriched in the yolk sac PK44 population compared with E10.0 AGM PK44 cells (Hou et al., 2020) were related to angiogenesis and vasculature development (Figure 5C). In comparison, those related to regulation of cell fate specification and mesodermal cell differentiation were enriched in the AGM PK44 population (Figure 5C). These data emphasize the distinct identities of PK44 cells from different sampling sites.

Similar to that from the AGM region, yolk sac PK44 cells could also be further separated into two subpopulations by unsupervised clustering, which were, respectively, annotated as endothelial- and hematopoietic-biased subpopulations according to their DEGs. *Nrp1*, *Tek*, *Cdh5*, *Gja5*, *Kdr*, were highly expressed in the endothelial-biased subpopulation of yolk sac PK44 cells (Shalaby et al., 1997; Nishikawa et al., 1998; Takakura et al., 1998; Simons and Eichmann, 2015), and the hematopoietic-biased subpopulation was featured by the obvious expression of *Kit*, *Adgrg1*, and transcription factor *Runx1* (North et al., 2002; Taoudi et al., 2005; Zilberberg et al., 2012; Swiers et al., 2013; Zhou et al., 2016; Hou et al., 2020) (Figures 5D,E). Gene Ontology terms related to angiogenesis were enriched in the endothelial-biased subpopulation, and ribosome biogenesis was enriched in the hematopoietic-biased subpopulation of yolk sac PK44 cells (Figure 5F). By further comparing the hematopoietic-biased subpopulations from the yolk sac and that from the AGM region, we detected a set of DEGs similar to those extracted from the comparison of PK44 cells between two sites (Figure 5B), including *Lyve1* and *Stab2*, which were relatively highly expressed in the former (Nonaka et al., 2007; Lee et al., 2016; Ganuza et al., 2018; Shvartsman et al., 2019) (Figure 5G). Considering the unambiguous repopulating capacity of the progenies of PK44 cells from both the yolk sac and AGM region (Figures 4F–H and Supplementary Figure 3A), these data together suggest the existence of spatially and molecularly distinct HECs with a multilineage hematopoietic potential.

Then, PK44 cells from the yolk sac and the AGM region in our previous dataset (Hou et al., 2020) were pooled for integrative analysis. Interestingly, PK44 cells from the yolk sac and AGM region were not initially distinguished by their sampling locations. Rather, they were clustered predominantly according to whether they presented endothelial- or hematopoietic-biased characteristics (Figure 5H). Featured by the relatively high expression of *Nrp1*, *Gja4*, and *Gja5*, the integrated endothelial-biased cluster was separated from the integrated hematopoietic-biased cluster, which was featured by *Adgrg1* and *Spi1* expression (Figures 5H,I). This finding further provides a molecular basis for the diverged potentials of PK44 cells from both the yolk sac and AGM region (Figures 4A,B).

## Discussion

In the present study, the existence of three molecularly different subpopulations within the AGM PK44 population were transcriptomically identified. Subsequently, the subpopulations with either endothelial- or hematopoietic-biased characteristics were functionally validated by using the marker combinations of Kit and CD93/CD146 within the PK44 population through flow cytometry index sorting. The finding complements the characteristics of heterogeneous subpopulations in HECs that were unknown before (Hou et al., 2020). Meanwhile, the concomitant presence of endothelial-hematopoietic dual potential cells in both endothelial- and hematopoietic-biased subpopulations suggests the PK44 population as a continuum for the EHT process.

Based on the functional validation, we further revealed a previously unknown HSC competence of HECs represented by the PK44 population in the yolk sac around the time of HSC emergence during embryogenesis. Unexpectedly, the three differentiation potentials such as that in AGM region PK44 cells were found within yolk sac PK44 cells. The capture of the endothelial-hematopoietic dual potential suggests that a part of PK44 cells in the yolk sac might be undergoing EHT.

Transcriptionally, two subpopulations characterized by endothelial or hematopoietic bias were separated by unsupervised clustering within yolk sac PK44 cells. Unexpectedly, when the yolk sac and AGM PK44 cells were pooled for integrative analysis, the cells gathered together according to their endothelial- or hematopoietic-biased characteristics rather than different embryonic sites, which provided a molecular basis for the diverged potentials of PK44 cells from both the yolk sac and AGM region.

Interestingly, unlike the AGM PK44 cells, the proportion of yolk sac PK44 cells with only hematopoietic potential at E9.5 was significantly higher than that at E10.0. A possible explanation is that part of the yolk sac PK44 cells differentiated into erythroid-myeloid progenitor cells, thus increasing the proportion of hematopoietic clusters. However, this hypothesis needs to be further verified by functional experiments. Our study also found that, the chimerism in the peripheral blood of adult recipient mice transplanted with progenies of AGM and yolk sac PK44 cells both peaked at 4 weeks post-transplantation and then decreased gradually, probably because most HSC-primed HECs have completed EHT by E10.5, and the remaining HECs, which could be obtained by our sorting strategy in the present study, only retain very limited potential to generate HSCs.

In summary, our study paves the way for further analysis of the developmental progress of HSCs and provides clues for the expansion of HSCs *in vitro*. In addition, a comprehensive understanding of the spatiotemporal and functional heterogeneity of HECs helps us to comprehend the process of hematopoietic development.

## Data Availability Statement

The scRNA-seq data of yolk sac PK44 cells have been deposited in NCBI’s Gene Expression Omnibus (GEO) with the accession number GSE173833. The scRNA-seq data of AGM PK44 cells have already been deposited in the GEO under accession number GSE139389.

## Ethics Statement

The animal study was reviewed and approved by the Academy of Military Medical Sciences (Fifth Medical Center of Chinese PLA General Hospital).

## Author Contributions

YL and BL designed the study. Y-QL performed cell sorting, culture, immunostaining, HSC transplantation assays with help from YN, SH, HW, CW, JW, JH, RY, JY, and GZ. TH performed scRNA-seq. YG and DL performed bioinformatics analysis. Y-QL, YL, BL, and SH wrote the manuscript and revision. All authors reviewed the manuscript.

## Conflict of Interest

The authors declare that the research was conducted in the absence of any commercial or financial relationships that could be construed as a potential conflict of interest.

## Publisher’s Note

All claims expressed in this article are solely those of the authors and do not necessarily represent those of their affiliated organizations, or those of the publisher, the editors and the reviewers. Any product that may be evaluated in this article, or claim that may be made by its manufacturer, is not guaranteed or endorsed by the publisher.

## Supplementary Material

Supplementary Figure 1 Functional experiments using index sorting. **(A)** Representative CD31 and CD45 immunostaining on the cultures of single PK44 cells from E10.0 AGM, showing typical morphologies regarding distinct differentiation potentials (left). Scale bars, 100 μm. Cell frequencies of each kind of potential within PK44 cells are shown (right). Data are from 20 independent experiments. **(B)** Cell numbers of each kind of potential in presumed subpopulations within E10.0 AGM PK44 cells. **(C)** Cell numbers of each kind of potential in sorted subpopulations within E10.0 AGM PK44 cells. **(D)** Expression of Kit, Flk1, CD304, CD47, and CD49d on the index-sorted single E10.0 AGM PK44 cells. Cells with different kinds of potentials based on *in vitro* functional evaluation (color dots) are mapped onto the reference FACS plots (gray dots). Data are from three independent experiments. The enlarged views of red boxes are shown below. **(E,F)** Column charts showing quantitative analyses of the differentiation potential of different presumed **(E)** or prospectively sorted **(F)** subpopulations as indicated. Data are means ± SD. The statistical significance of differences was determined using Pearson’s chi-squared test. ^∗^*P* < 0.05; ^∗∗^*P* < 0.01, ^∗∗∗^*P* < 0.001.

Supplementary Figure 2 Expression of surface markers in the index-sorted single E9.5 yolk sac PK44 cells. Cells with different kinds of potentials based on *in vitro* functional evaluation (color dots) are mapped onto the reference FACS plots (gray dots). Data are from three independent experiments. The enlarged views of red boxes are shown below.

Supplementary Figure 3 Multiorgan and multilineage repopulations at 16 weeks post-transplantation of primary recipients transplanted with the progenies of E10.5 AGM and yolk sac PK44 populations. **(A)** Representative FACS plots showing donor-derived (CD45.1^+^CD45.2^+^) myeloid (Ly6G^+^/Mac-1^+^), B lymphoid (B220^+^), and T lymphoid (CD3^+^) cells in peripheral blood and multiple hematopoietic organs. **(B,C)** Representative FACS plots of immunophenotypic HSCs (Lin^–^Kit^+^Sca-1^+^Flk2^–^CD34^–^) in bone marrow of the primary recipients transplanted with the progenies of AGM (upper) and yolk sac (lower) PK44 cells.
