# Supplementary figures and images for: Spatiotemporal and Functional Heterogeneity of Hematopoietic Stem Cell-Competent Hemogenic Endothelial Cells in Mouse Embryos
Source: Front Cell Dev Biol. 2021 Aug 11;9:699263. doi: 10.3389/fcell.2021.699263 (PMC8385538; doi:10.3389/fcell.2021.699263)

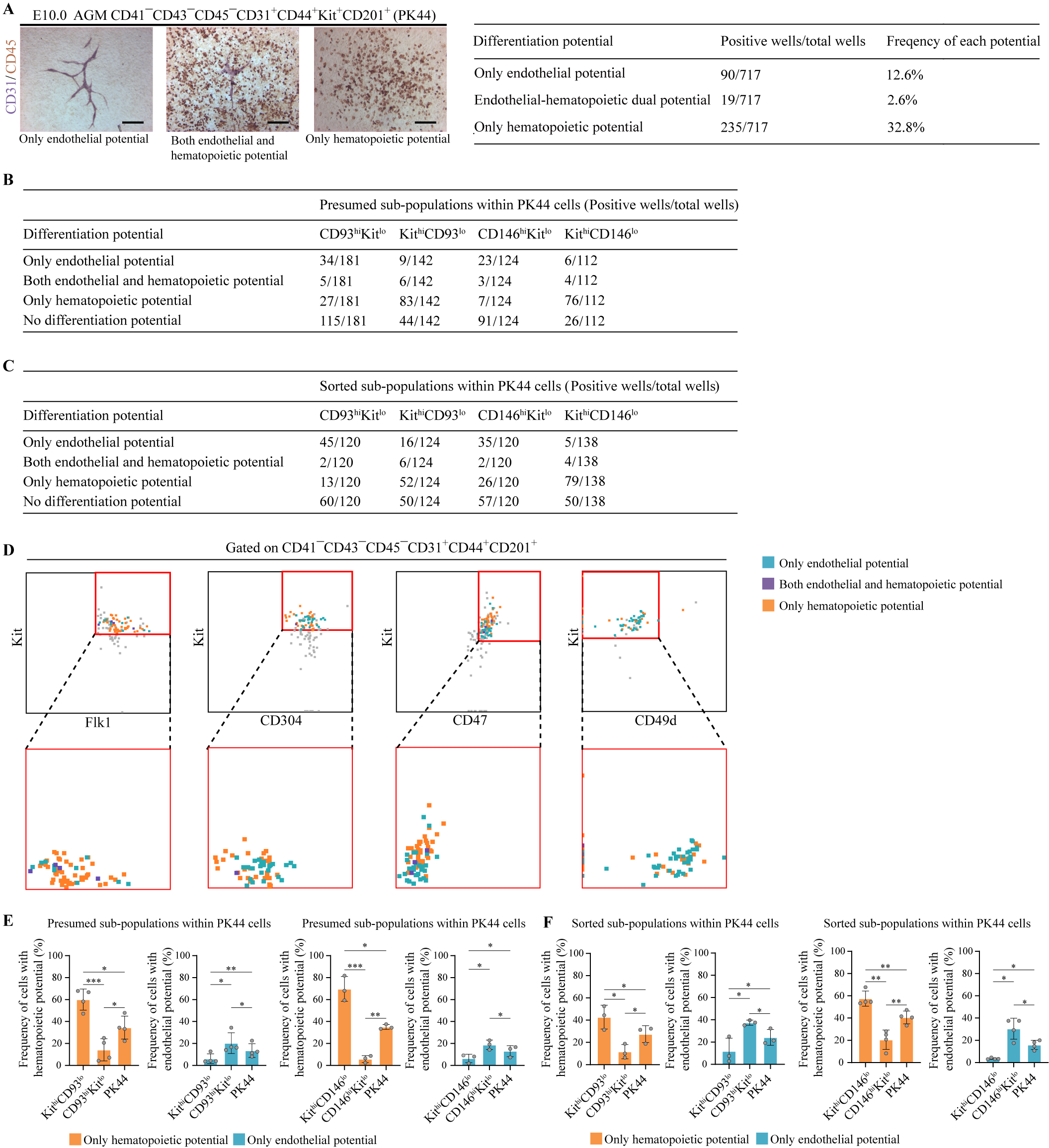

Supplement: Supplementary Figure 1 — Functional experiments using index sorting. (A) Representative CD31 and CD45 immunostaining on the cultures of single PK44 cells from E10.0 AGM, showing typical morphologies regarding distinct differentiation potentials (left). Scale bars, 100 μm. Cell frequencies of each kind of potential within PK44 cells are shown (right). Data are from 20 independent experiments. (B) Cell numbers of each kind of potential in presumed subpopulations within E10.0 AGM PK44 cells. (C) Cell numbers of each kind of potential in sorted subpopulations within E10.0 AGM PK44 cells. (D) Expression of Kit, Flk1, CD304, CD47, and CD49d on the index-sorted single E10.0 AGM PK44 cells. Cells with different kinds of potentials based on in vitro functional evaluation (color dots) are mapped onto the reference FACS plots (gray dots). Data are from three independent experiments. The enlarged views of red boxes are shown below. (E,F) Column charts showing quantitative analyses of the differentiation potential of different presumed (E) or prospectively sorted (F) subpopulations as indicated. Data are means ± SD. The statistical significance of differences was determined using Pearson’s chi-squared test. ∗P < 0.05; ∗∗P < 0.01, ∗∗∗P < 0.001. [file Image_1.TIF]

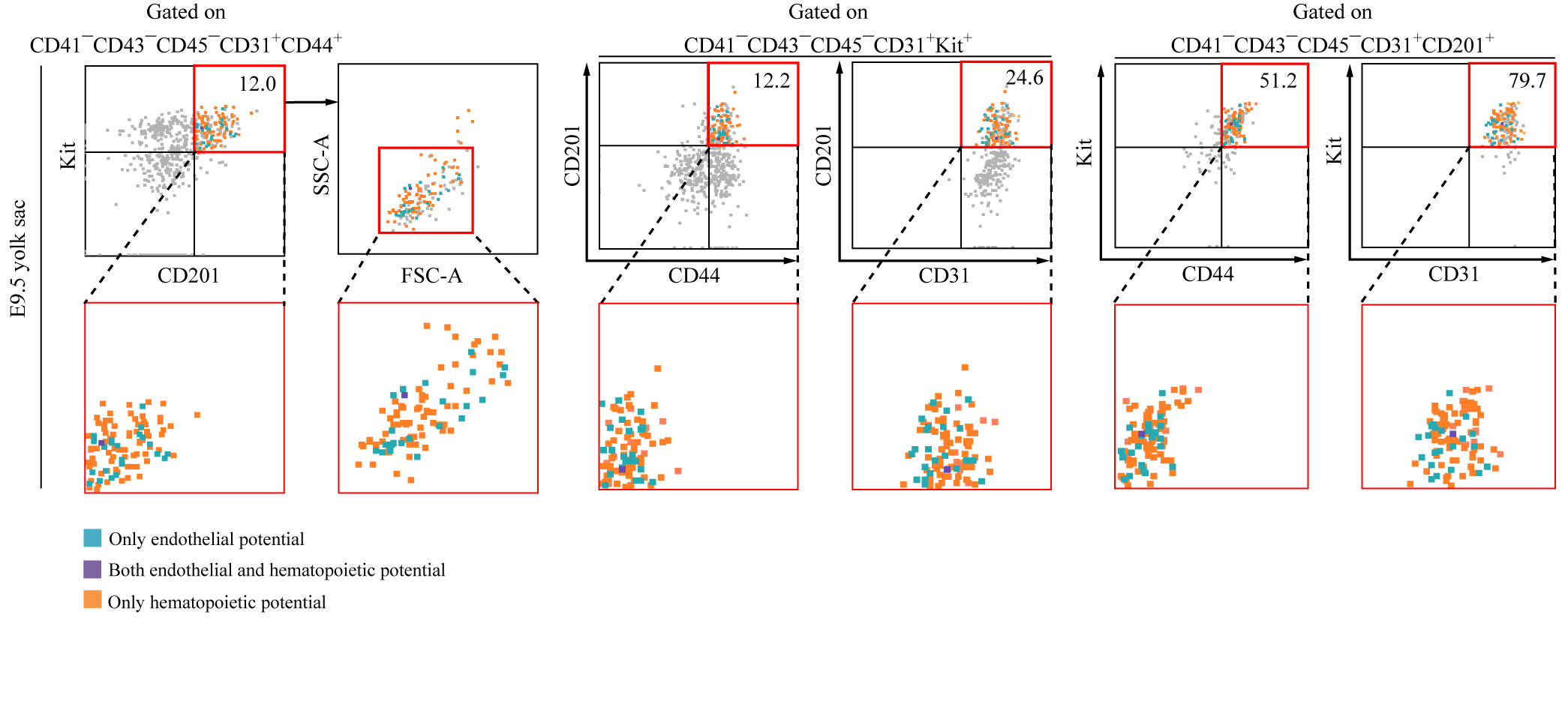

Supplement: Supplementary Figure 2 — Expression of surface markers in the index-sorted single E9.5 yolk sac PK44 cells. Cells with different kinds of potentials based on in vitro functional evaluation (color dots) are mapped onto the reference FACS plots (gray dots). Data are from three independent experiments. The enlarged views of red boxes are shown below. [file Image_2.TIF]

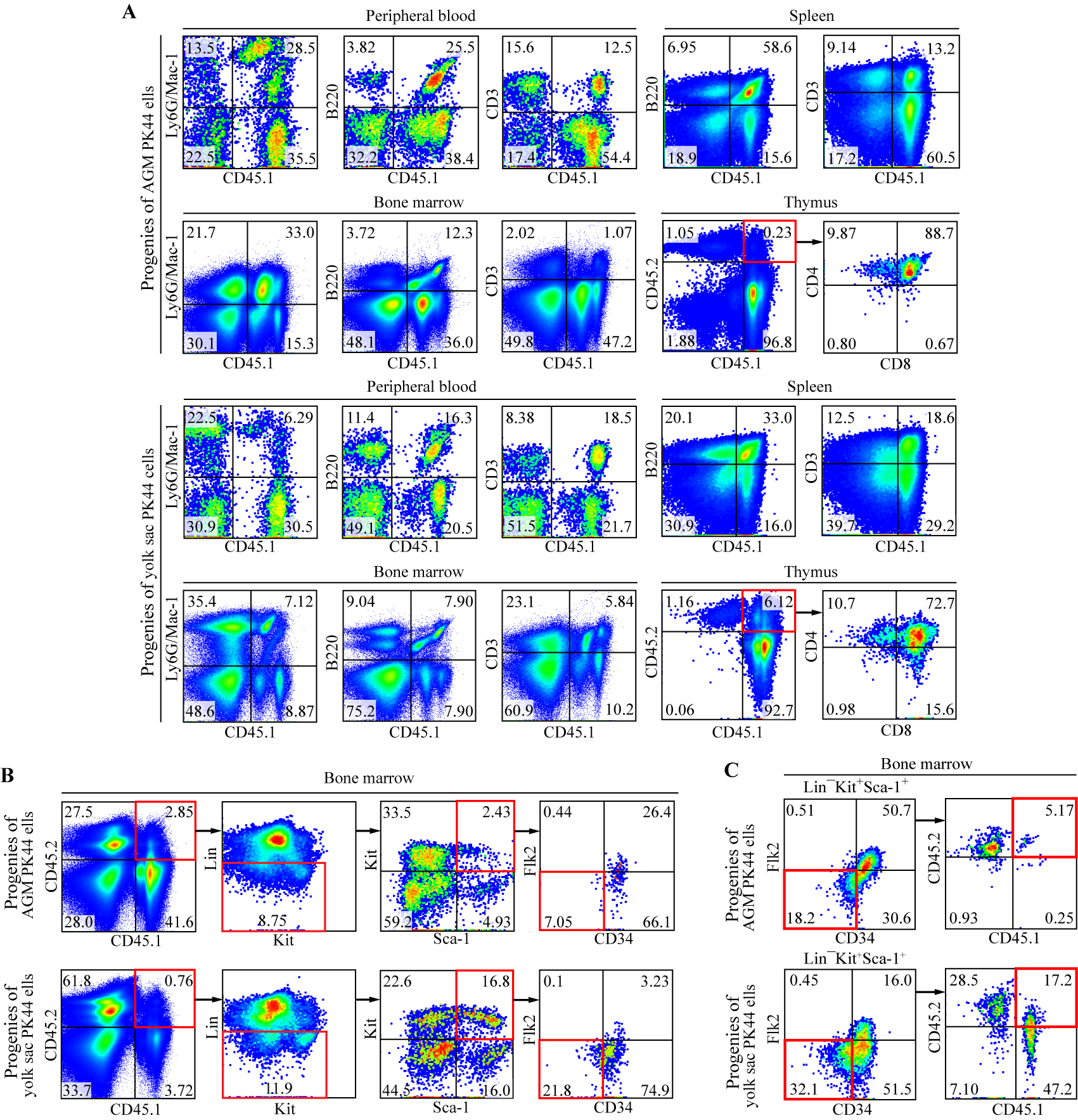

Supplement: Supplementary Figure 3 — Multiorgan and multilineage repopulations at 16 weeks post-transplantation of primary recipients transplanted with the progenies of E10.5 AGM and yolk sac PK44 populations. (A) Representative FACS plots showing donor-derived (CD45.1+CD45.2+) myeloid (Ly6G+/Mac-1+), B lymphoid (B220+), and T lymphoid (CD3+) cells in peripheral blood and multiple hematopoietic organs. (B,C) Representative FACS plots of immunophenotypic HSCs (Lin–Kit+Sca-1+Flk2–CD34–) in bone marrow of the primary recipients transplanted with the progenies of AGM (upper) and yolk sac (lower) PK44 cells. [file Image_3.TIF]
